# Network-Wide Adaptive Burst Detection Depicts Neuronal Activity with Improved Accuracy

**DOI:** 10.3389/fncom.2017.00040

**Published:** 2017-05-31

**Authors:** Inkeri A. Välkki, Kerstin Lenk, Jarno E. Mikkonen, Fikret E. Kapucu, Jari A. K. Hyttinen

**Affiliations:** ^1^BioMediTech Institute and Faculty of Biomedical Sciences and Engineering, Tampere University of TechnologyTampere, Finland; ^2^Department of Computer Science and Information Systems, University of JyväskyläJyväskylä, Finland; ^3^Pervasive Computing, Faculty of Computing and Electrical Engineering, Tampere University of TechnologyTampere, Finland

**Keywords:** burst detection, neuronal networks, microelectrode arrays, burst synchrony, network classification

## Abstract

Neuronal networks are often characterized by their spiking and bursting statistics. Previously, we introduced an adaptive burst analysis method which enhances the analysis power for neuronal networks with highly varying firing dynamics. The adaptation is based on single channels analyzing each element of a network separately. Such kind of analysis was adequate for the assessment of local behavior, where the analysis focuses on the neuronal activity in the vicinity of a single electrode. However, the assessment of the whole network may be hampered, if parts of the network are analyzed using different rules. Here, we test how using multiple channels and measurement time points affect adaptive burst detection. The main emphasis is, if network-wide adaptive burst detection can provide new insights into the assessment of network activity. Therefore, we propose a modification to the previously introduced inter-spike interval (ISI) histogram based cumulative moving average (CMA) algorithm to analyze multiple spike trains simultaneously. The network size can be freely defined, e.g., to include all the electrodes in a microelectrode array (MEA) recording. Additionally, the method can be applied on a series of measurements on the same network to pool the data for statistical analysis. Firstly, we apply both the original CMA-algorithm and our proposed network-wide CMA-algorithm on artificial spike trains to investigate how the modification changes the burst detection. Thereafter, we use the algorithms on MEA data of spontaneously active chemically manipulated *in vitro* rat cortical networks. Moreover, we compare the synchrony of the detected bursts introducing a new burst synchrony measure. Finally, we demonstrate how the bursting statistics can be used to classify networks by applying k-means clustering to the bursting statistics. The results show that the proposed network wide adaptive burst detection provides a method to unify the burst definition in the whole network and thus improves the assessment and classification of the neuronal activity, e.g., the effects of different pharmaceuticals. The results indicate that the novel method is adaptive enough to be usable on networks with different dynamics, and it is especially feasible when comparing the behavior of differently spiking networks, for example in developing networks.

## Introduction

Neuronal networks are often studied using microelectrode arrays (MEAs). A MEA consists of a number of electrodes recording the extracellular electrical activity, including the action potentials, of the neuronal network at multiple locations simultaneously. Therefore, MEAs are especially useful in studies considering neuronal networks instead of single cells (Heikkilä et al., 2009; Johnstone et al., 2010). The neuronal networks are typically characterized by their spiking activity, of which an elemental part is bursting (Wagenaar et al., 2006).

Bursting is typically described as “periods of dense spiking separated by quiescent periods.” Often bursts are determined using thresholds for the minimum spike rate in a burst or the maximum interspike intervals in the burst. In the simplest case, these thresholds are fixed (Chiappalone et al., 2005), but they can also be determined based on the properties of the spike train based on e.g., the mean ISI, spike rate, or the distribution of the ISIs (Wagenaar et al., 2006; Mazzoni et al., 2007).

The burst detection methods that determine the bursts based on the data at hand can be called adaptive algorithms, as they are able to adapt the burst definition to the data. The main advantage of the use of adaptive algorithms is the ability to process multiple types of activity using the same algorithm. On the other hand, at the same time the burst definition becomes different for various types of activity.

For example, the cumulative moving average (CMA) algorithm (Kapucu et al., 2012) calculates the burst and tail thresholds for bursts based on the skewness of the CMA of the interspike interval (ISI) distribution. The advantage of the algorithm is its adaptability: it can automatically detect bursts on various types of spike trains, especially on developing stem cell derived neuronal networks, where the activity varies greatly over time and between networks. On the other hand, the algorithm has some major limitations, as it has also been stated by Cotterill et al. (2016): In sparse spike trains, the algorithm results in very sparse and long bursts. Also, *post-hoc* screening proposed by Kapucu et al. (2012) and Cotterill et al. (2016) to correct the erroneously detected bursts by using statistical analysis may become cumbersome as the bursts have different definitions on all the spike trains. Additionally, the method only considers one spike train at a time, but for evaluating bursting as an activity of a widely-distributed network, including the activity of multiple electrodes in the analysis may give further insight. On the other hand, another adaptive algorithm by Pasquale et al. (2010) considered network-wide bursts; however, the algorithm mainly requires a clear separation between inter- and intra-burst intervals in the ISI histograms which usually cannot be encountered in developing neuronal cells (Kapucu et al., 2012; Cotterill et al., 2016). Thus, an enhanced method which both considers the network-wide bursting and applicability for developing neuronal cells is required.

Mainly, burst detection algorithms are based on time series data which is recorded from single electrodes (Chiappalone et al., 2005; Pasquale et al., 2010; Kapucu et al., 2012). However, network-wide bursting has been mostly analyzed based on the computation of network-wide firing rates recorded from all the recording sites. Accordingly, an instantaneous increase in the total firing activity, i.e., a synchronized firing on some number of channels would be considered as network bursts by those algorithms, without considering whether the considered channels show bursting activity separately or not (van Pelt et al., 2004; Mazzoni et al., 2007; Raichman and Ben-Jacob, 2008). Pasquale et al. (2010) considered the network bursts differently by first detecting bursts in separate channels and calculating cumulative burst events for network wide from the calculated bursts; then, a network burst is considered as a burst of burst events. Wagenaar et al. (2006) considered bursts only if they appear simultaneously across several electrodes. Accordingly, synchronization of the network has been also evaluated by means of simultaneously occurred events, i.e., simultaneous spikes and bursts across different recording sites.

In addition to the above studies based on network burst assessment, there are several other methods estimating network synchrony. For example, event synchrony (Quiroga et al., 2002), mutual information (Gray, 1990), and transfer entropy (Schreiber, 2000) utilizes simultaneous network spiking and CorSE (Kapucu et al., 2016a) utilize simultaneous changes in the signal complexity for estimating synchrony; however, these methods are not merely depending on the existence of simultaneous bursts, thus beyond the scope of this paper.

In this work, we aim to inspect if including the whole network activity to the adaptive burst detection enhances the network-wide burst analysis. Therefore, we extend the CMA algorithm to include the whole MEA activity when determining the burst threshold. We also show how the same modified algorithm method can be used to pool data for analysis: for example, by combining the spike trains of one or multiple channels from multiple measurements within the same study.

To demonstrate the behavior of the burst detection algorithms, we first apply the algorithms on artificial data resembling *in vitro* MEA data to show how the burst detection differs between the algorithms. After that, we use the algorithms on MEA data from spontaneously active and chemically treated rat cortical neurons to show, how the network-wide burst detection is more consistent defining the bursts in multiple measurements, and therefore more suitable for network-wide analysis. Moreover, we compare the synchrony of the detected bursts by analyzing how simultaneous they are. Additionally, we demonstrate how the burst statistics from both the original and modified algorithms can be used to classify networks using the k-means algorithm.

## Materials and methods

### Artificial data

To demonstrate and evaluate the different CMA algorithms (described in Section Burst Detection), we applied the methods on artificial spike trains resembling *in vitro* MEA measurements. The artificial data consisted of sets of 60 spike trains from which we calculated the burst rate, burst duration, mean ISI inside bursts, and mean ISI outside bursts.

Each of the spike trains was constructed as follows. First, the burst periods were determined: The start times for the bursts were randomly chosen according to the burst rate of the channel (5, 10, 15, or 20 bursts per minute). To prevent the bursts from overlapping, the burst period start times were adjusted so that the distance between two consecutive burst periods was at least 2 times the burst period length of each channel. Then, the burst period lengths were chosen normally distributed around the mean burst period length of the channel (150, 325, or 500 ms). The spike times were defined according to the mean ISIs of the channel: The ISIs were drawn from the exponential distributions corresponding to the mean ISIs of the channel, depending on the momentary bursting state of the channel. In one artificial dataset, all the 60 channels had the same mean ISI in the non-burst periods. Depending on the channel, the mean ISI in the bursting periods was 10–100 times lower than the non-burst mean ISI. This way, all the artificial datasets had electrodes with varying spike- and burst-rates, and burst durations.

To surrogate a series of MEA measurements, we made six artificial datasets with different non-bursting state mean ISIs with 1,000, 2,000, …, 6,000 ms. This resembles a series of experiments, where an activity-increasing drug is applied on the network in different amounts.

**Figure 1:**
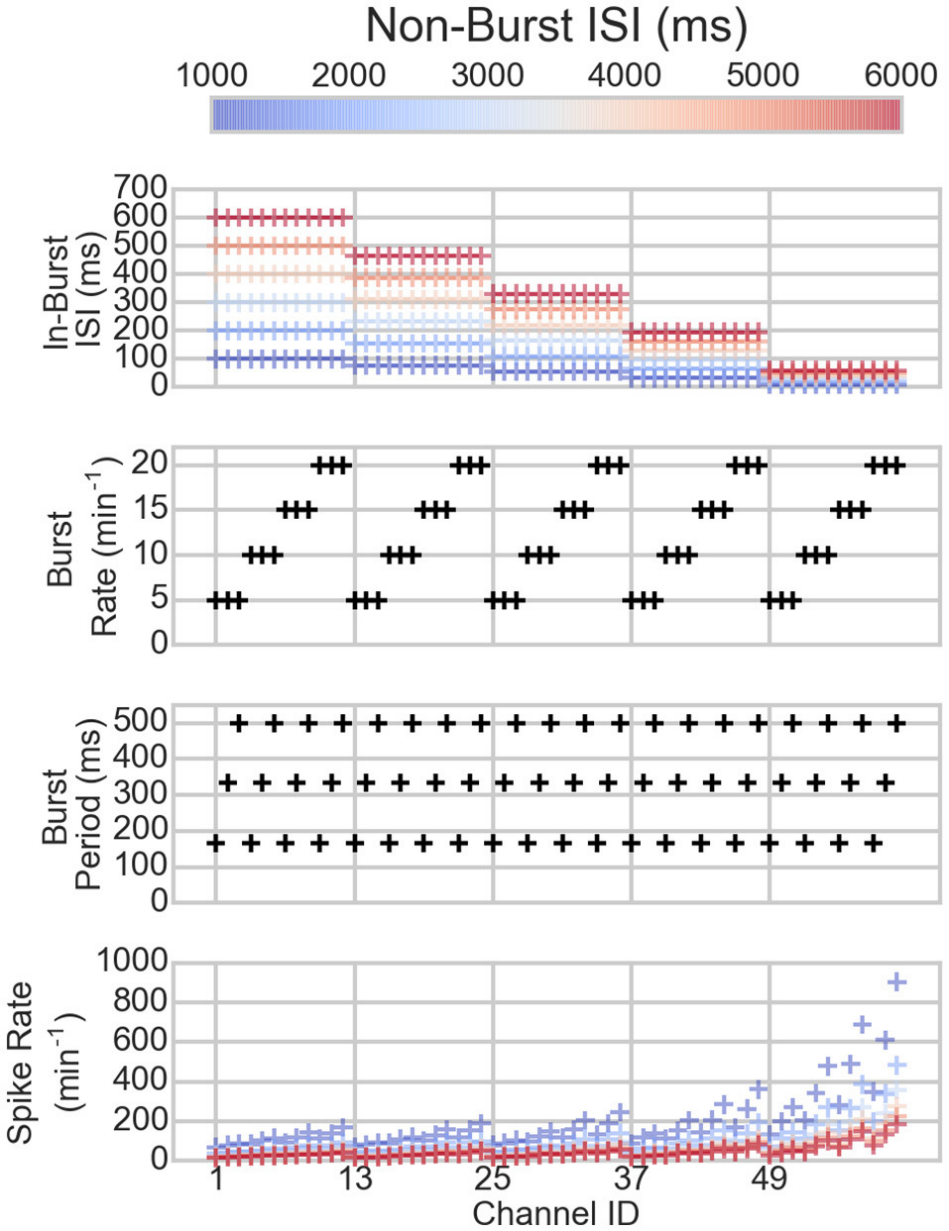
**The parameters of the channels of the artificial data (three topmost panels) and the resulting spike rates (bottom panel)**. On the x-axis are the different channels. Each mark corresponds to one channel in one dataset. The colors correspond to the non-burst ISIs in the dataset, hence, the same colored markers belong to the same dataset. The black marks show the values of the channels that are not changed between the different datasets.

The bursting and spiking parameters of the artificial spike trains are shown in Figure 1 along with the resulting mean spike rates. The spike rates vary depending on the spiking and bursting parameters of the spike train: the lower the ISIs and the longer the bursts, the higher the spike rate, as can be expected.

### MEA data

The biological MEA data was recorded from spontaneously active and chemically treated rat cortical neurons. All the experimental procedures regarding animals were conducted between 2007 and 2008 and were implemented in accordance with European Commission Recommendation 2007/526/EC on the accommodation and care of animals used for experimental and other scientific purposes. Protocols related to animal experiments were approved by the Institutional ethics committee (Freiburg University). In short, prefrontal cortical tissue from zero-day-old Wistar rats was first dissected, then cells were enzymatically dissociated (Tetzlaff et al., 2010) and finally cultured on polyethylene-imine (Sigma-Aldrich) coated MEAs with 60 TiN electrodes of 30 μm diameter and either 200 μm spacing or 500 μm spacing (Multi Channel Systems, Reutlingen, Germany). The culture medium was composed of MEM supplemented with 5% heat-inactivated horse serum, 0.5 mM L-glutamine and 20 mM glucose (all compounds from Gibco Invitrogen). Cultures were stored in a standard cell incubator and two thirds of the medium was replaced twice per week. Animals were treated according to the Freiburg University's and German guidelines on the use of animals in research. Neuronal density after the first day *in vitro* (DIV) was estimated from phase contrast images at ≈1,250 neurons/mm^2^.

Neuronal activity was recorded inside a dry incubator housed with a custom made liquid cooler (Mikkonen et al., 2008). The recorded signal was amplified (gain 1,100, 1–3,500 Hz) and sampled at 25 kHz/12 bit (MEA 1060-BC, Multi-Channel Systems, Reutlingen, Germany). One electrode served as internal reference. Spontaneous activity of each MEA was recorded for 10 min. Additional recordings were conducted with pharmacological manipulations, 30 min for each measurement file. Spikes were detected with five times the standard deviation threshold and waveforms were recorded from −1 to 2 ms. Only spike times and spike wave forms were stored on hard drive.

Cultures were treated with selective blockage of two excitatory glutamate dependent synaptic transmission receptors α-amino-3-hydroxyl-5-methyl-4-isoxazole-propionate (AMPA) or N-methyl-D-aspartic acid (NMDA) by 2,3-dihydroxy-6-nitro-7-sulfamoyl-benzo[f]quinoxaline-2,3-dione (NBQX), and (2*R*)-amino-5-phosphonovaleric acid; (2*R*)-amino-5-phosphonopentanoate (AP5) respectively. Furthermore, inhibitory GABA_A_ blockage was induced by competitive antagonist of GABA_A_ receptors bicuculline. Glutamate antagonists block the excitatory synaptic transmission while GABA_A_ antagonist blocks inhibitory synaptic transmission. Both AMPA and NMDA receptors are glutamatergic blockers emphasizing the variable nature of excitation: NMDA receptors require depolarization of the post-synaptic membrane prior to receptor channel activation/opening. Pharmacological manipulation was performed as follows. Increasing concentrations of antagonists/ agonists were directly pipetted into cell culture medium on MEAs. The pipetted volumes were 5, 10, 20 μL for all reagents and additionally 50 μL for bicuculline. After re-pipetting high concentrations (generally 40 or 50 μM) two pharmacological agents were co-introduced in some cultures in order to better discriminate specific AMPA, NMDA, and GABA_A_ effects. Additional concentrations were acquired by re-pipetting. After pharmacological manipulations, the cell cultures were washed twice with fresh medium and returned to the incubator. The number of recordings varied between MEAs. The detailed information of the number of recordings as well as used chemicals and concentrations are shown in Table 1.

**Table 1:** **The chemicals and their concentrations used for stimulus in MEA experiments**.

**MEA 1**	**Concentration (μM)**									
Bicuculline		5	10							
NBQX				1	2	5	10	20	50	100
Count	3	4	45	2	2	2	1	1	33	3
**MEA 2**	**Concentration (μM)**									
NBQX		2	5	10	20					
Count	2	1	2	2	32					
**MEA 3**	**Concentration (μM)**									
AP5		2	2	5	10	20	20	50		
NBQX			2	2	2	2	20	20		
Count	1	1	1	1	25	36	1	1		
**MEA 4**	**Concentration (μM)**									
AP5		2	5	10	20					
Count	2	1	1	1	1					
**MEA 5**	**Concentration (μM)**									
NBQX		5	10	20						
count	1	1	1	1						

*The first number of the counts indicates the number of measurements of spontaneous activity. On MEA 3, AP5, and NBQX were applied at the same time*.

In total, we analyzed the activity recorded from five MEAs with different pharmacological treatments, see Table 1. The channels which showed more than 3,000 spikes per minute were excluded from the analysis, because by visual inspection, the very high spike rate was seen to be because of false detected spikes, not a high spike rate of the neural network.

### Burst detection

The bursts on both the artificial and MEA data were detected using adaptive algorithms handling one or multiple spike trains at a time. We used the CMA algorithm introduced earlier (Kapucu et al., 2012), which calculates the burst threshold for each channel separately (Section The Original CMA). We extended the algorithm to handle multiple spike trains simultaneously (Section The Modified Multi-CMA algorithm) and applied this modified algorithm on the spike trains from one measurement, or multiple measurements at different time points.

#### The original CMA

The original CMA algorithm by Kapucu et al. (2012) is the base of our network wide burst detection methods. The original algorithm calculates the ISI histogram and its skewness value from a spike train. The calculated skewness value is stored to be used for the calculation of an ISI threshold for the putative bursts. Next, the CMA of the ISIs is calculated as a function of ISI bins in the histogram as in Kapucu et al. (2012):

Let *y*_*i*_, *i* = 1, …, *N*, with *N* the total number of ISI bins, be the spike count in the *i*th ISI bin. The value of the cumulative sum of the histogram *CH*_*I*_ at the *I*th, *I* ≤ *N*, ISI bin is defined as

(1) $$\begin{array}{r} CH_{I}=\sum_{i=1}^{I}y_{i} \end{array}$$

The corresponding CMA is given by

(2) $$\begin{array}{r} CMA_{I}=\frac{1}{I}\sum_{i=1}^{I}y_{i} \end{array}$$

whose maximum, *CMA*_*m*_, is reached at the *m*th ISI bin, and

(3) $$\begin{array}{r} m=\underset{k=1,\;\ldots ,N}{\text{arg max}}\left(\frac{1}{k}\sum_{i=1}^{k}y_{i}\right) \end{array}$$

This point represents the maximum that the average spike count reaches: in other words, for the ISI values beyond this point, the average count starts to decrease. Accordingly, *CMA*_*m*_ is used as a critical point for the calculation of the thresholds of intra-burst ISIs together with the skewness value of the ISI histogram. For that, a skewness dependent factor, α_1_, where 0 < α_1_ < 1, is defined and the threshold of intra-burst ISIs is set to α_1_ *CMA*_*m*_. In addition, burst related spikes are considered and included in the bursts by using another skewness dependent factor α_2_, where α_2_ < α_1_ and α_2_ >0. Accordingly, the threshold for burst related spikes is set as α_2_·*CMA*_*m*_. We used the same skewness and alpha relations as suggested in Kapucu et al. (2012) in this study. After detecting the putative burst and burst related spikes, burst related spikes which are not following or followed by a burst are omitted. Also, the bursts which are closer to each other than the threshold calculated for the burst related spikes, α_2_·*CMA*_*m*_, are merged together.

#### The modified multi-CMA algorithm

We extended the above described original CMA-algorithm to consider the activity of multiple channels simultaneously, instead of the activity in only one spike train, and thus enabling to analyze a certain area or the entire network activity at once. The modified algorithm is here called *multi-CMA*.

**Figure 2:**
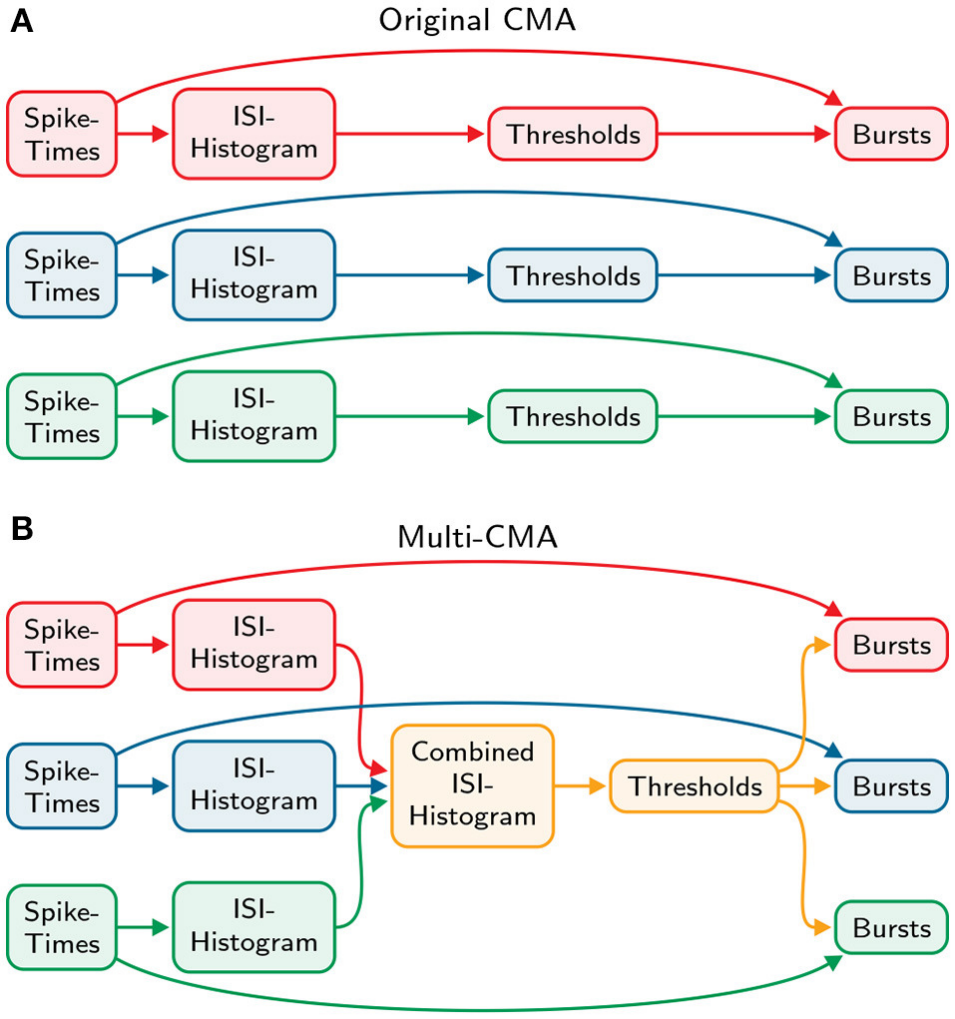
**Comparison of the original and multi-CMA algorithms. (A)** The original CMA algorithm calculates the ISI-histogram individually for each spike train. Then the burst- and tail thresholds are calculated separately for each ISI-histogram. These thresholds are then used to detect bursts in each spike train. **(B)** The multi-CMA algorithm starts also with calculating the ISI-histograms of the spike trains. Thereafter, it combines the ISI histograms of all chosen spike trains to be processed to one histogram. Then the burst- and tail thresholds are calculated based on this combined histogram similarly to the original algorithm described above. These thresholds are then used to detect bursts in all the spike trains individually.

Multi-CMA starts similarly to the original algorithm by calculating the ISI-histograms of the individual spike trains, *y*_*q, i*_, after which the combined histogram, *CH*_*I*_ is calculated as follows

(4) $$\begin{array}{r} CH_{I}=\sum_{q=1}^{Q}\sum_{i=1}^{I}y_{q,\;i} \end{array}$$

where *q* = 1, …, *Q*, with *Q* the number of processed spike trains combined in one histogram, e.g., *Q* = 60, if histogram is calculated from all the channels of a 60-channel MEA measurement. Then, *CMA*_*I*_ is calculated as

(5) $$\begin{array}{r} CMA_{I}=\frac{1}{I}\sum_{q=1}^{Q}\sum_{i=1}^{I}y_{q,\;i} \end{array}$$

and *m*th ISI bin where *CMA*_*m*_ is located is calculated as

(6) $$\begin{array}{r} m=\underset{k=1,\;\ldots ,N}{\text{arg max}}\left(\frac{1}{k}\sum_{q=1}^{Q}\sum_{i=1}^{k}y_{q,\;i}\right) \end{array}$$

similarly to the original-CMA algorithm. Finally, the burst- and tail thresholds are determined identically to the original algorithm described above. These thresholds are then used to detect bursts in all the spike trains individually. Both the original and the modified algorithm are demonstrated and compared in Figure 2.

The multi-CMA can be used to detect bursts on any set of spike trains, for example an area of a network, the entire network or spike trains from different time points or even measurement sets, depending on the needs of the analysis. The idea is to choose the spike trains on which one wants to apply the same criteria for burst detection. In this study, we used four different versions of the algorithm:

- *Original-CMA*: each channel in each measurement was analyzed individually
- *Network-CMA*: all the channels in each MEA measurement were analyzed together
- Single-*channel-CMA*: the spike trains from one channel in multiple measurement time points on the same MEA were analyzed together
- *MEA-CMA*: all the electrodes of one MEA in multiple measurement time points were analyzed together

The first algorithm is the original-CMA and the three latter algorithms are based on the multi-CMA approach. The Matlab code for multi-CMA has been developed to be applied straight forward on any time series data, and is publicly freely available in the Matlab Central File Exchange.

### Burst synchrony

We compared the synchrony of the bursts detected by the different CMA algorithms described above by analyzing how simultaneous the detected bursts are on different channels. The algorithms described above determine the start and end times of the bursts for each spike train. From these burst time stamps, we calculated how many channels on the MEA are bursting at each time point. This gives a temporal “burst signal” which describes the network bursting recorded by the MEA: When there are no channels bursting, the signal is zero; and when multiple channels show bursts at the same time, the burst signal gets high values. Thus, if many channels of the MEA are bursting synchronously, the signal should have clear peaks and possibly rhythmicity.

To characterize if the network is bursting synchronously, we calculated the variance-to-mean ratio for the burst signal, which we call here the *burst synchrony*. This value describes, how dispersed the values of the burst signal are. The larger this ratio is, the more synchronous the bursting is: For a synchronously bursting network, the mean is close to zero, but the variance is still high, as during the network bursts there are multiple neurons bursting.

### Clustering of bursting and spike data

The MEA measurements were clustered according to the burst statistics obtained with the different burst detection algorithms. The used statistics were: spike rate (in spikes per minute), burst rate (in bursts per minute), burst duration (in seconds), the number of spikes in a burst, burst/spike ratio, ISI in burst (in milliseconds), number of bursting channels, spike rate on the bursting channels (in spikes per minute), burst rate on the bursting channels (in bursts per minute), burst/spike ratio on the bursting channels, and burst synchrony as defined in Section Burst Synchrony. For each measurement, the mean value of the statistics was calculated. Each of the statistical measures was then normalized by dividing by the maximum value of the statistic. The mean values that could not be calculated (e.g., when there were no bursting channels in the measurement, the spike rate on bursting channels cannot be calculated), were set to an arbitrary value of –99.

The measurements were clustered using the k-means algorithm with correlation as the distance metric (Bora and Gupta, 2014). To find the optimal number of classes, we ran the k-means algorithm with between 2 and 15 classes. To minimize the variability of the results for each class, the algorithm ran 100 times. The best number of classes was chosen as the one having the highest mean silhouette value of all classes.

## Results

### Burst detection

We used four burst detection algorithms, as described in the previous section, to detect bursts on artificial spike trains and biological MEA data. The algorithms were original-CMA (single channel, single time point), network-CMA (multiple channels, single time point), channel-CMA (single-channel, multiple time points), and MEA-CMA (multiple channels, single time point).

#### Artificial data

We firstly demonstrated the four different burst detection algorithms with artificial data resembling MEA measurements. Each artificial dataset consisted of 60 spike trains, so-called virtual channels, with different ISIs during the bursts, burst rates and burst durations. All together, we had 6 artificial datasets corresponding to a series of MEA measurements.

**Figure 3:**
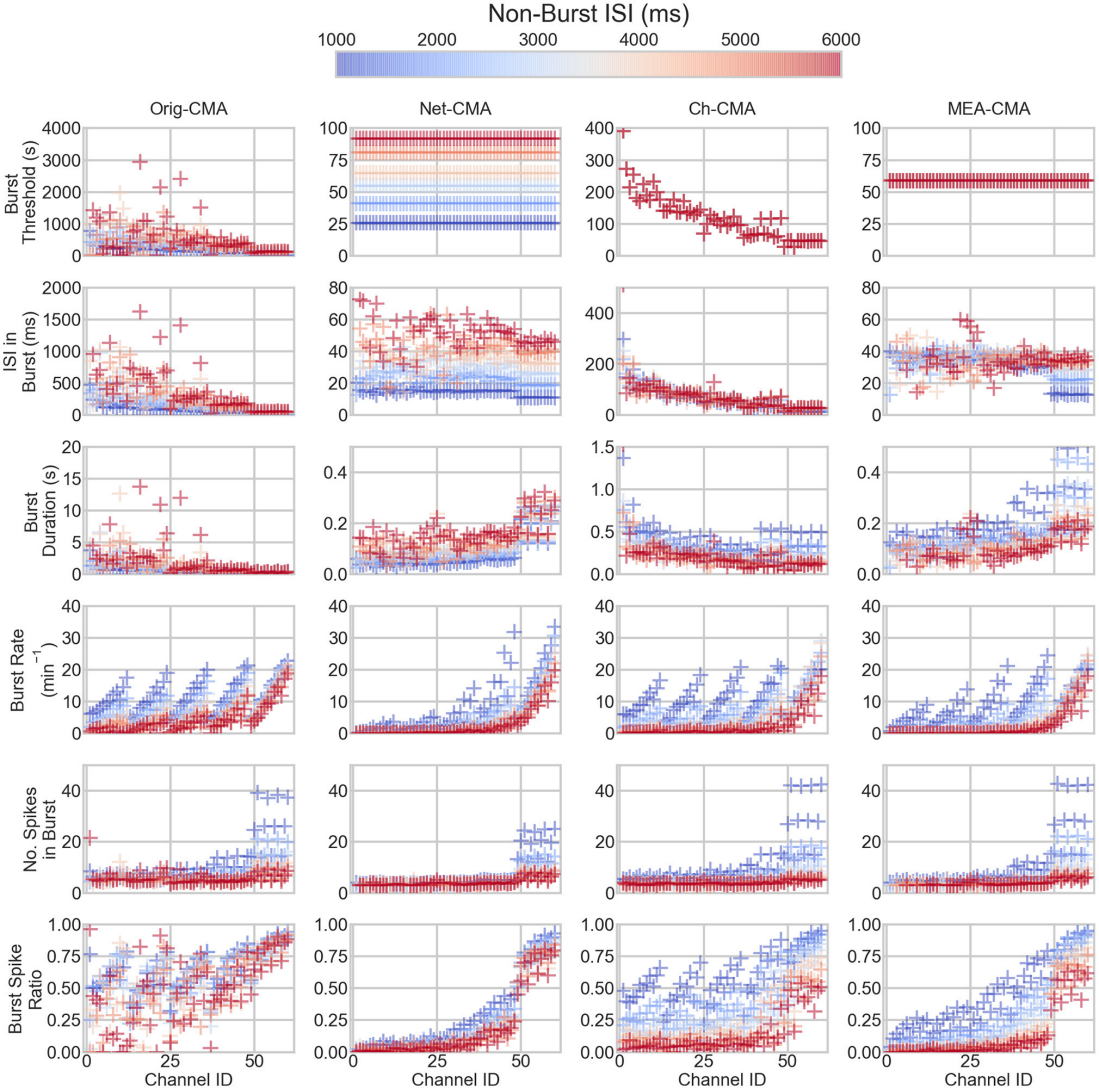
**The bursting statistics of the artificial data**. The panel columns correspond to different CMA-algorithms and the rows the different statistics of the bursting. Each mark corresponds to one channel in one dataset. The colors correspond to non-burst ISI in the dataset. Hence, the same colored markers and mean lines belong to the same dataset. The ID is the channel number. For the parameters of the channels, compare to Figure 2. Please note the different y-axes limits on the same bursting statistic.

The bursting statistics detected with different algorithms are shown in Figure 3, which displays the statistics according to the channel ID, so that the bursting statistics can be compared with the burst parameters shown in Figure 1. The first row of the figure shows the burst thresholds determined with the different burst detection algorithms. It can be seen that the original-CMA is very adaptive in determining the burst thresholds: Depending on how dense the spiking on the electrode is (see also Figure 1), the burst threshold is defined accordingly, which results in very high burst thresholds for sparsely spiking electrodes, even multiple seconds. The other CMA algorithms, which consider multiple spike sequences simultaneously, define stricter burst thresholds. This is because the combined ISI-histogram in the multi-CMA has more ISIs from the actively spiking channels than from the sparsely spiking. The network-CMA defines much lower thresholds than the original-CMA, which excludes many bursts on the sparsely spiking channels. The channel-CMA gives quite variable thresholds for different channels so that it can still detect spikes on also the less active channels, but also here the very extreme values of the original-CMA are greatly reduced. The MEA-CMA gives only one threshold, which is in the same range as the thresholds of the network-CMA.

The burst threshold greatly determines the ISIs in the bursts, shown on the second row in Figure 3. Here, it can be seen how the original-CMA detects many sparse bursts that are excluded by the multi-CMAs. The burst duration (row 3) also correlates with the burst threshold and ISI in burst: sparser bursts also typically last longer.

The row 4 of Figure 3 shows the burst rates on the artificial spike trains. It can be seen how the original-CMA is very adaptive, and the burst rates correlate very well with the nominal burst rates shown in Figure 1. This is also seen in the burst rates detected with the channel-CMA, but here the most sparsely spiking datasets also have lower burst rates. However, the network-CMA and MEA-CMA detect only very few bursts on the sparsely spiking electrodes.

Also, the number of spikes in a burst (row 5) tends to increase as the spike rate increases. However, the strict burst thresholds of the network- and MEA-CMAs keep the burst spike number lower than in the original- and channel-CMAs. The burst spike ratio (bottom row) shows how well the original-CMA can adjust to the spike sequence: The burst spike ratio is quite high for most of the channels. However, the network-wide CMAs show much lower burst spike ratios for the sparsely spiking electrodes and high for the densely spiking.

Table 2 shows the standard deviations in the simulated measurements. These values demonstrate, how the standard deviations detected with the multi-CMAs are lower than with the original-CMA. This demonstrates that the burst statistics vary less in a network, when detected with multi-CMA, as expected.

**Table 2:** **The standard deviations of the burst statistics of the simulated data**.

	**Orig-CMA**	**Net-CMA**	**Ch-CMA**	**MEA-CMA**
Burst threshold (ms)	379.94	0^*^	69.36	0^*^
ISI in burst (ms)	235.31	5.82	47.16	7.03
Burst duration (s)	2,488	60	146	72
Burst rate (1/min)	4.74	6.07	5.12	5.21
No. spikes in burst	3.75	2.81	3.12	3.78
Burst spike ratio	0.21	0.28	0.20	0.26

Each value is the mean of the standard deviations of the individual simulated measurements. The two zeros marked with “*” are real zeros, because there is no deviation in the network as only one limit is applied in the whole network.

#### MEA data

**Figure 4:**
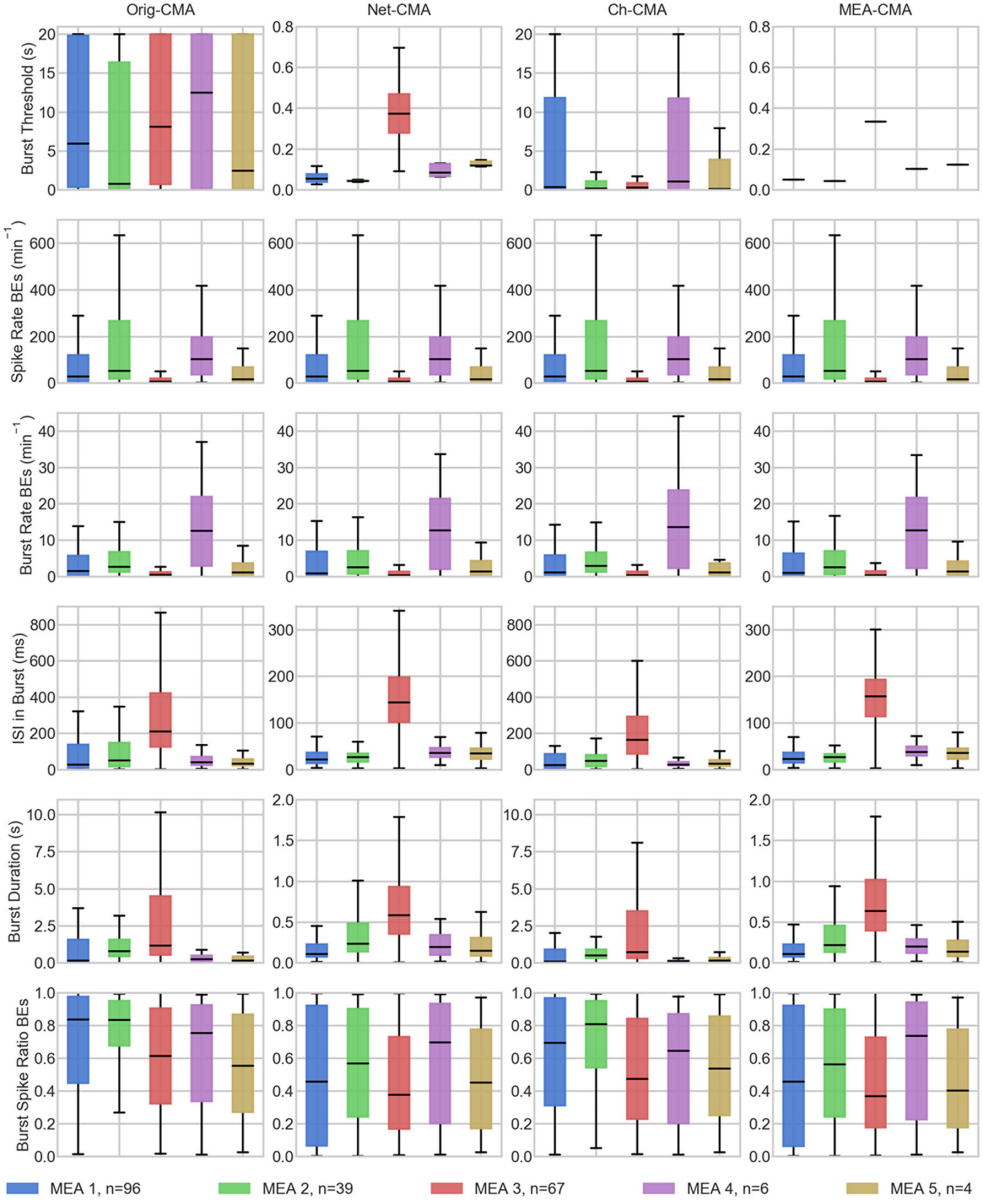
**The bursting statistics of the MEA data**. The columns are the different CMA algorithms, and the rows the different statistics of bursting. *n* in the legend shows the number of measurements on the MEA. BE stands for bursting electrodes. Please note the different y-axes limits on the same bursting statistic.

**Figure 5:**
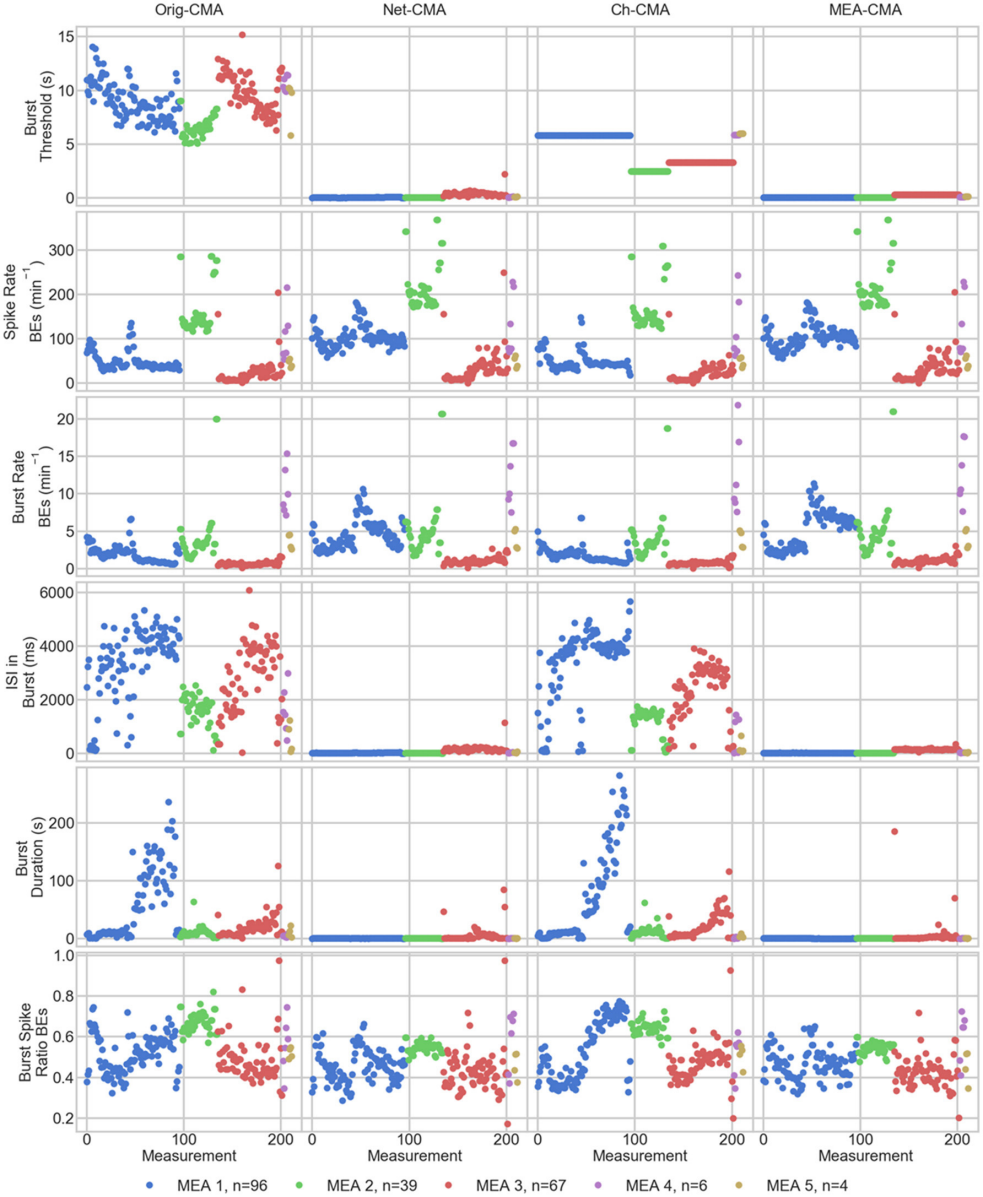
**The measurement means of the bursting statistics of the MEA data**. Each mark corresponds to a mean in one measurement. The columns are the different CMA algorithms, and the rows the different statistics of bursting. The colors correspond to different MEAs, and n shows the number of measurements on the MEA. On the x-axis are the different measurements. BE stands for bursting electrodes.

The statistics of the bursts detected on the MEA data are shown in Figure 4. Figure 5 shows the mean values of each dataset and for each of the four CMA-algorithms separately. The second to last row of Figure 5 shows the number of the *bursting electrodes*, i.e., electrodes on which bursts were found in each measurement using the different algorithms, on the y-axis.

The first row of Figures 4, 5 show the burst thresholds determined by the different burst detection methods. As can be seen in Figure 4, the original-CMA determines burst thresholds in a wide range, with very high values, even multiple seconds, on many electrodes. This also makes the mean values in the MEA-measurements very high. The network-CMA gives much stricter thresholds: all the thresholds are here less than 1 s. Also, it can be seen how the thresholds for the measurements from the same MEA have similar thresholds. The thresholds determined with the channel-CMA are similar to the original-CMA, but the ratio of the very high thresholds is a bit lower. The MEA-CMA gives one threshold for each MEA, about the same range as the network-CMA.

In Figure 5, as it can be expected from the burst thresholds, the network-CMA does not find bursts on as many electrodes as the original-CMA that adapts to each spike train individually. Also, MEA-CMA finds bursts on roughly the same number of channels as the network-CMA. Channel-CMA finds bursts on more electrodes than MEA-CMA. Additionally, the channel-CMA finds bursts on more channels than the network- and MEA-CMA, but not as many as the original-CMA.

The spike rates of the bursting electrodes (in row two of Figures 4, 5) show that the channels on which multi-CMAs do not find bursts are typically sparsely spiking: The number of bursting electrodes with low spike rate is much higher, when using the original-CMA than any of the multi-CMAs, as expected. Also, the mean spike rate on the bursting electrodes is typically higher with the network-CMA than the original-CMA. Additionally, the burst rates (in row 4) show how the network- and MEA-CMAs typically find multiple bursts on the bursting channels, and the burst rates are often higher compared to the original- and channel-CMAs.

The burst threshold influences greatly the ISIs in bursts (row 4 in Figures 4, 5). The original-CMA detects many bursts that have very long ISIs in the bursts, around 10 s. However, with the network-CMA the maximum mean ISIs in bursts are in hundreds of milliseconds. Also, here it can be seen that the bursts detected with the network-CMA are similar in the measurements from the same MEA. Also, MEA-CMA finds only the dense bursts, but the channel-CMA detects also many sparse bursts. The ISIs in burst also greatly affects the duration of the detected bursts (row 5). The bursts with longer ISIs are often also long in duration.

The burst spike ratio (row 6) shows how the original-CMA can adjust to the individual spike trains, and the number of electrodes with high burst spike ratio remains high even on the sparsely spiking channels. The burst spike ratio is similar, when using the channel-CMA, but with the network- and MEA-CMAs there are much more of those bursting channels, where the burst spike ratio is very low.

The synchrony measure (bottom row in Figure 5) shows how the bursts detected with the network- and MEA-CMAs are often more synchronized than the ones detected with the original- and channel-CMAs. Also, the synchrony values of one MEA are closer to each other when using the network- and MEA-CMAs than with original- and channel-CMAs.

### Clustering of MEA data

**Figure 6:**
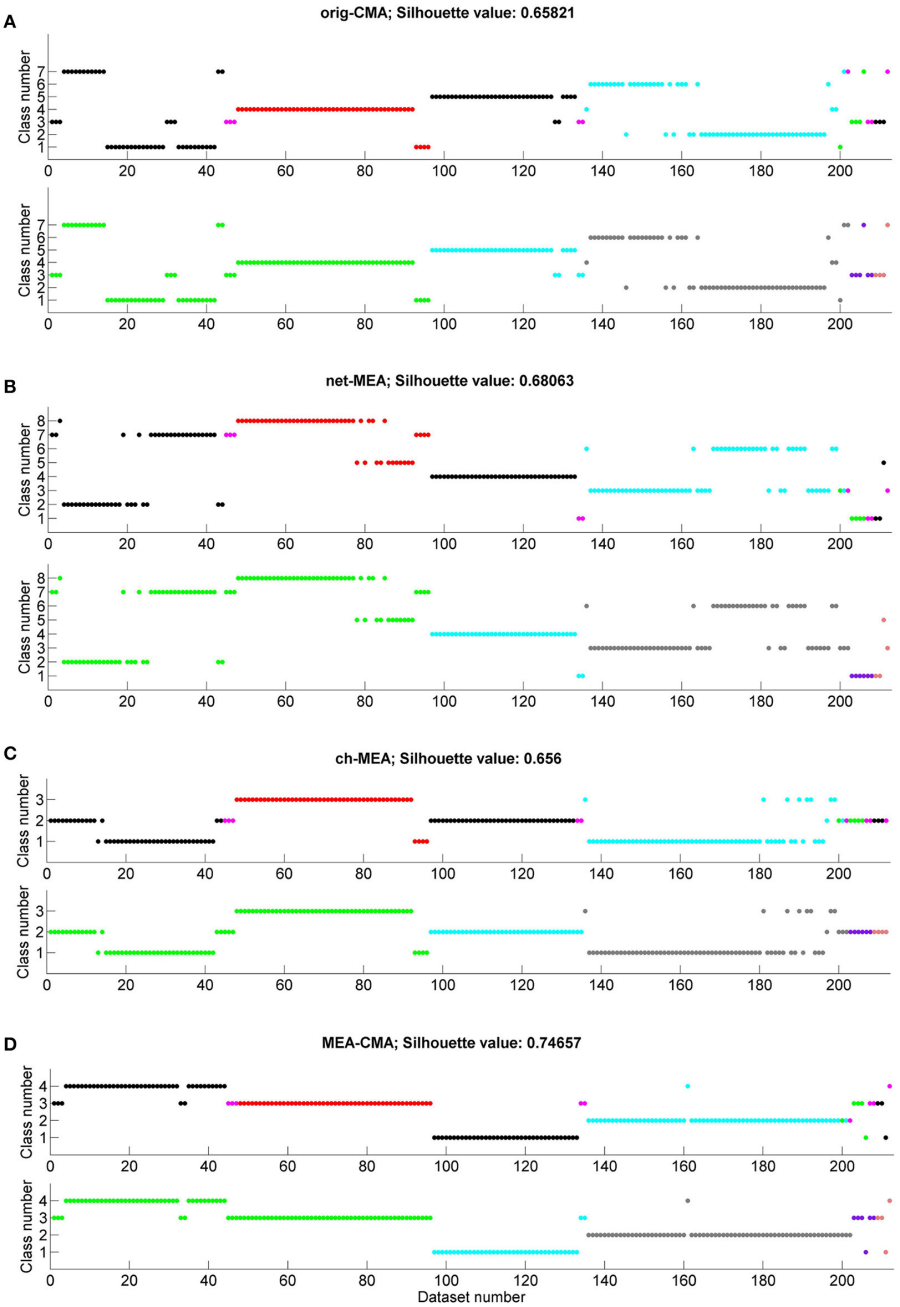
**Clustering of the MEA datasets according to the applied substances and individual MEAs**. Each graph shows on the x-axis the number of the dataset (compare with Table 1) and on the y-axis the class number obtained by the k-means clustering. The color code shows either the spontaneous activity (spont)/ applied substance(s) (NBQX, BIC=bicuculline, AP5, AP5+NBQX; in the upper graph in each subfigure **(A–D)** or the individual MEAs (lower graph in each subfigure **A–D**). On top of each subfigure the silhouette value is presented. Classification results are shown for **(A)** original-CMA, **(B)** network-CMA, **(C)** channel-CMA, and **(D)** MEA-CMA.

We applied k-means to the statistics on the spiking and bursting parameters calculated with each of the four CMA variants. Figure 6 shows for each CMA algorithm the determined class number for the applied substances and each individual MEA, respectively. We color-coded the results to see if we can differentiate (a) the substance and (b) the MEAs by its spike and burst behavior. The average silhouette value was calculated for each clustering. The best performing CMA was defined as the one where most or all datasets of one substance or MEA were in the same class. NBQX was best classified when the burst features where calculated with the MEA-CMA (see Figure 6D). In more detailed analysis, we observed that k-means can distinguish between the concentrations 20 and 50 μM NBQX (figure not shown). For bicuculline (BIC) the channel-CMA worked the best (Figure 6C), for AP5 the channel-CMA and network-CMA (Figures 6C,B), for AP5+NBQX the MEA-CMA (Figure 6D), and for spontaneous activity channel-MEA (Figure 6C). As expected, k-means performs best with MEA-CMA to distinguish the activity of each MEA (see Figure 6D).

## Discussion

In this study, we inspected if network-wide burst detection can be enhanced by including the whole network activity to the adaptive burst detection in MEA analysis. The previously published CMA-algorithm focused more on the single channel bursting; thus, it is limited to population activity which is detectable from one electrode, also it provides information of the local network, e.g., burst participation of different types of spikes (Kapucu et al., 2016b). On the other hand, MEA recordings have potential to include the information from wider distributed networks and a different type of analysis is invoked for the assessment of network-wide bursts. However, the adaptation of burst rules separately for which channel renders the comparison of entire network activity or activity from different time points difficult.

Therefore, we extended an adaptive burst detection method, the CMA-algorithm using just single channel to adapt the rule (Kapucu et al., 2012), to analyze multiple spike trains simultaneously and derive the adaptive burst rule using this network activity separately. The spike trains used for the new analysis can be, for example, from a specific area of a network, or one whole MEA. The same algorithm can also be used to pool data from multiple measurements recorded in different time points and derive a single burst rule from the entire data.

In this study, we compared four different adaptive burst detection algorithms: the original-CMA, which analyses one spike train at a time, and three types of multi-CMAs analyzing multiple spike trains simultaneously. For multi-CMA, the spike trains we either all the electrodes of one measurement (network-CMA), all the measurements of one channel (channel-CMA), or all the spike trains of a MEA pooled together (MEA-CMA).

### Artificial data

The methods were first compared using artificial spike trains. The artificial data consisted of sets of differently spiking and bursting spike trains resembling a series of MEA measurements to illustrate the differences of the burst detection results with different CMA-algorithms. These results (Figure 3) showed how the original CMA, which analyzes each spike train individually, is most adaptive, as expected. When the bursts are determined based on only one single spike sequence, the algorithm adapts so that it find bursts also on the very sparse spike sequences, but also can handle the denser sequences. This issue has been discussed in previous papers as well (Kapucu et al., 2012; Cotterill et al., 2016). Because of this adaptive nature of the algorithm, the parameters of burst definitions has potential to become very diverse in MEA thus, the comparison of the burst statistics inside the network and between different networks becomes complex. However, we and others have been successful on using such kind of statistics to assess the network changes e.g., under different pharmacological manipulations (Mack et al., 2014; Kapucu et al., 2016b).

When comparing the bursting statistics in Figure 3 with the nominal parameters of the spike trains in Figure 2, it can be seen how the burst statistics follow the nominal burst parameters of the artificial channels. Especially the original-CMA adapts to each spike train separately, and therefore the detected burst rates follow strongly the nominal burst rates. However, the burst rates detected with the network-CMA do not follow the nominal parameters so well. This was as expected as the burst definition depends on the activity of the whole network, which excludes many bursts on the sparse spike trains as the simulated individual spike trains do not have common network origin as they are individually generated. Also, the standard deviations of the burst statistics, shown in Table 2, demonstrate, how the statistics are less varying, when bursts are detected with multi-CMA-algorithms, which suggests that these statistics obtained with multi-CMAs better describe the whole network activity than the statistics based on the original-CMA algorithm.

Moreover, pooling the one channel data from different time points (channel-CMA) removes many of the very high burst thresholds, and the long and sparse bursts with them. However, the network-CMA and especially the MEA-CMA uniform the definition of a burst and give reasonable thresholds for bursts in differently behaving datasets, as can be seen in Figure 3.

### MEA data

The four developed burst detection methods were applied on MEA data from spontaneous active and chemically treated neuronal cultures. The results showed once more how the original-CMA algorithm, which analysis one channel at a time, is very adaptive and can be used to detect bursts on different spike trains. However, it resulted even in sparse spike trains sometimes sparse and long bursts. On the other hand, the network wide bursts detection was able to adapt to the different behavior of individual MEAs, which also resulted in variable burst parameters, although less variable than the original CMA algorithm.

The statistics from the bursting show how the network-wide burst detection provides more robust analysis, especially when analyzing sparse spike trains. As seen in Figure 4 from the spike rates of the bursting electrodes, the original-CMA often finds bursts also on the sparsely spiking channels, where the network-CMA does not. This is a consequence of the resulting different burst thresholds given by the different algorithms. The original algorithm is able to adapt to the sparse spiking and gives very high thresholds. However, when multiple spike trains are analyzed simultaneously, the more active electrodes have more spikes, and hence there are more of the shorter ISIs and the thresholds for the burst detection become lower. The stricter burst thresholds in turn exclude the very sparse and long bursts. However, if the networks behave differently, and as seen from Figure 5, the network-CMA can adapt on multiple types of spike sequences by keeping the burst detection thresholds still adaptive.

None of the pharmacological treatments alone was able to totally abolish bursting within the dissociated cortical culture. Therefore, the remaining unblocked receptors types took over and dominated the retained activity or the concentrations of the used glutamatergic and gabaergic blockers were not able to entirely terminate the receptor function. Our results show that regardless of the receptor type, selective pharmacology altered burst patterns in a systematic and pharmacological agent, or blocker, specific way. This can be seen in, how the different pharmacological treatments are clustered together based on the bursting statistics of the networks.

In general, in the multi-CMAs, the channels with dense spiking affect more the burst thresholds than the less-active channels, since the ISIs are shorter in spike trains with a higher spike rate, and therefore the shape of the ISI-histogram has more weight on the shorter ISIs.

### Burst synchrony

Our bursting synchrony method showed how the burst criteria is of great importance on analyzing the networks. The synchrony of the bursts in the networks was determined based on the number of the simultaneously bursting channels and the variance-to-mean ratio for the burst signal, which we call here the burst synchrony. As can be seen in Figure 4 bottom row, the bursts detected with the network wide methods are more synchronous and the CMA based on individual spike train analysis resulted more non-synchronous bursting highlighting the importance of the algorithm selected for the burst definition. As the bursts are generally considered to be network wide, this clearly demonstrates how the network wide algorithms can provide better data that describe the network instead of CMA based on a single channel.

### Clustering of MEA data

The clustering results show that substances are best classified when single-channel-CMA was used to detect bursts. As can be seen in Figure 6C, k-means is able to separate the bicuculline datasets in class 3. Class 2 contains mainly NBQX datasets. Some of the NBQX datasets and almost all AP5+NQBX are mixed in class 1. While bicuculline is a GABA_A_ receptor antagonist and thus blocks the inhibitory synaptic activity, AP5 and NBQX are both glutamate receptor antagonists and thus block the excitatory transmission. This indicate that the network contributing the local single channel has independence that is described best by the single-channel-CMA and it is useful when comparing the same small network pharmacological responses.

As expected, different MEAs are well-separated when the MEA-CMA was used (Figure 6D). The burst activity of MEA 1 is divided into two classes whereas the datasets belonging to class 4 are mainly NBQX data and datasets belonging to class 3 bicuculline data. The cluster algorithm is able to distinguish between treatments with NBQX (MEA 2) and AP5+NBQX (MEA 3).

The mean silhouette value is largest for the MEA-CMA meaning that the datasets are assigned very well to the corresponding clusters. Repeated analyses of the MEAs resulted in better clustering. In general, and not surprisingly, all methods worked better in distinguishing gabaergic from glutamatergic pharmacological manipulation. Blocking GABA_A_ receptors caused a relative increase in excitatory transmission, but at the same time the decreased inhibition reduced synchrony. Interestingly, both single-channel-CMA and MEA-CMA could distinguish the different glutamatergic excitation pathways thus providing new insight on how bursts are formed in dissociated cortical cultures. NMDA receptor activation requires depolarization of the post-synaptic membrane and is thus been considered important for bursting and potentiation of synapses (Fung et al., 2016). AMPA receptors, on the other hand, are activated regardless of the polarization level and thus facilitate the depolarization required for NMDA receptor activation. Further research is needed to fully explore the mechanisms of and the contribution of different neurotransmitters in burst formation in small networks represented by the single channel or in larger networks of the entire MEA. However, this result highlight the usefulness of both locally adaptive burst detection and the MEA wide adaptation.

## Conclusion

Here, we developed an adaptive bursting definition and analysis framework called multi-CMA. According to our hypothesis and the results the main use of multi-CMA is to analyze all the spike trains in one MEA measurement at once so that the entire network behavior is included in the analysis, not only the spike train of a single electrode. This concept unifies the burst criteria on all the spike trains of the network. Moreover, the methods ranging from single spike train to multi-CMA can also be used to pool different datasets for analysis, depending on the hypothesis and need of the analysis, for example comparing of drug responses of a channel, spike trains from a MEA in developing network from multiple measurements, or even from arbitrary sets of electrodes or time points representing differently behaving areas in a MEA.

Moreover, the parameters derived from network-wide analysis provides good tool to analyze network bursts that will reflect the general behavior in a network that is not available in channel-specific burst analysis. The resulting bursts would be less variant comparing to the parameters derived from each separate channel. Consequently, such more robust network parameters would enable more reliable references for the comparisons, and assessment of the changes in the general network behavior for a variety of studies. Furthermore, the developed simple burstiness analysis provided an excellent measure of the synchrony of the network and provides with the MEA-CMA a set of tools to MEA wide network analysis.

In conclusion, the proposed modification of the adaptive burst definition algorithm provides means to tune the burst analysis to the hypothesis and analysis need in hand. It can be used to unify the burst definition in the whole network or in the set of data improving the assessment and classification of the network to be analyzed. On the other hand, the modified algorithm is still adaptive and can be utilized on variably behaving networks such as developing networks. This makes the set of new multi-CMA algorithms very versatile, it is feasible when comparing the behavior of networks during the development, toxicology, e.g., under chemical manipulations. The single channel CMA is especially useful in comparing the e.g., drug responses of the small network represented by the single channel. Moreover, the network level adaptation provides insights on the general assessment of network dynamics during network development and adaptation to stimulus or learning networks. Thus, the methods developed provide a powerful set of tools for MEA data analysis and can be adapted to a variety of needs and uses.

## Author contributions

IV made the burst detection and statistical analysis. KL did the classification of the datasets. FK developed and prepared the Matlab codes of the original CMA algorithm. JM conceived and designed the experiments and performed the experiments. All authors contributed reagents/materials/analysis tools and to the writing of the manuscript. All authors gave final approval for publication. All authors read and approved the final manuscript. All authors agree to be accountable for all aspects of the work in ensuring that questions related to the accuracy or integrity of any part of the work are appropriately investigated and resolved.

### Conflict of interest statement

The authors declare that the research was conducted in the absence of any commercial or financial relationships that could be construed as a potential conflict of interest.
